# Anthropometric determinants of change of direction speed in youth soccer players: A cross-sectional study using decision tree regression and machine learning approach

**DOI:** 10.1097/MD.0000000000046164

**Published:** 2025-11-21

**Authors:** Akram Fray, Yassine Negra, Halil İbrahim Ceylan, Younes Hachana, Valentina Stefanica, Mevlut Yildiz, Ahmed Attia

**Affiliations:** a University of Manouba, Higher Institute of Sport and Physical Education of Ksar-Saïd, Research Laboratory (LR23JS01) “Sport Performance, Health & Society,” Tunis, Tunisia; b Faculty of Sport Sciences, Ataturk University, Erzurum, Türkiye; c Department of Physical Education and Sport, Faculty of Sciences, Physical Education and Informatics, National University of Science and Technology Politehnica Bucharest, Pitesti University Center, Pitesti, Romania; d Coaching Sciences, Faculty of Sports Sciences, Mugla Sitki Kocman University, Mugla, Türkiye.

**Keywords:** anthropometric profiling, biological maturity, change of direction speed, decision tree regression, machine learning, talent identification, youth soccer players

## Abstract

This study aimed to determine the contribution of classic anthropometric features in young soccer players to predict their speed of preplanned change of direction performance using explainable artificial intelligence methodologies. Fifty-one young soccer players (age: 11.88 ± 1.38 years, body mass: 38.25 ± 7.89 kg) from a professional youth soccer academy were recruited. Several anthropometric features, including calf girth, leg length, sitting height, thigh length, and skinfold thicknesses, were employed following standardized protocols. Players’ change of direction speed was assessed using the 505-test. Biological maturity was estimated using the Mirwald equation. Five machine learning algorithms were implemented, with decision tree regression (DTR) selected as the optimal approach. Model validation employed leave-one-out cross-validation. The 505-test demonstrated excellent reliability (ICC = 0.86, SEM = 3.54%). Using the DTR model, anthropometric features accurately predicted change-of-direction speed performance (*P* < .05). The DTR model achieved superior predictive accuracy (R^2^ = 0.843, RMSE = 0.133, MAE = 0.111), explaining 84.3% of the variance in performance. DTR analysis revealed that demographic and anthropometric features, including sitting height and leg length, significantly affected change of direction speed performance (overall Gini importance > 0.5). Key predictors included maturity offset (16.3%), age (16.8%), leg length (15.8%), sitting height (13.6%), and knee height (10.5%). Youths who are more mature and have shorter sitting heights achieved superior change-of-direction speed performance. These findings underscore the importance of considering anthropometric and maturity characteristics in male youth soccer players to support talent identification, providing evidence-based frameworks for development programs.

**Key Points**

- This study used decision tree regression to predict the change of direction speed in youth soccer players based on anthropometric variables.
- The model explained 84.3% of performance variance, identifying maturity offset, leg length, and sitting height as key predictors.
- A 505-test was employed and shown to be highly reliable (ICC = 0.86, SEM = 3.54%).
- These findings support evidence-based, maturity-sensitive talent identification in youth sports.
- The machine learning approach offers interpretable insights for practitioners and coaches.

## 1. Introduction

Change of direction speed discriminates elite from subelite soccer players across youth populations and represents a practically relevant parameter for talent identification and selection processes.^[1–3]^ During competitive match play, professional soccer players execute rapid directional movements every 2 to 3 seconds, accumulating over 1000 directional changes per game.^[4]^ This frequency and intensity of directional changes underscores the critical importance of change of direction speed as a performance-determining factor in modern soccer, establishing a clear rationale for understanding the morphological determinants of this fundamental capability.^[5]^

Anthropometric measures refer to quantitative assessments of the human body, including dimensions such as height, weight, limb lengths, and body composition (e.g., fat mass and lean mass).^[6,7]^ These measures are widely used across various fields for purposes such as health monitoring, assessing growth and development, evaluating nutritional status, and testing physical fitness.^[6,7]^ In clinical and public health contexts, anthropometric data help identify growth abnormalities, obesity risk, and developmental delays.^[8]^ In physical education and sports science, they are utilized to evaluate body proportions and physical readiness for specific tasks or activities.^[9,10]^ In recent years, anthropometric assessments have also gained prominence in the domain of athletic talent identification. This application is based on the premise that specific physical characteristics may predispose individuals to superior performance in specific sports or activities.^[10,11]^

The relationship between human physical characteristics and sports performance has sustained continuous research interest, with anthropometric characteristics identified among key factors for early athletic talent identification.^[6,7]^ Recent investigations by Negra et al^[1]^ revealed significant associations between change of direction speed assessed via the 505 CoD test mean speed (m·s^−1^) and critical morphological variables, including fat mass, sitting height, and maturity offset in youth male soccer players (mean age: 12.4 ± 1.3 years). These findings establish quantitative relationships between readily measurable anthropometric parameters and directional change performance capabilities. The biomechanical mechanisms underlying these anthropometric associations reflect fundamental principles of human movement during directional transitions. Athletes with lower centers of mass, typically characterized by shorter sitting heights relative to total stature, demonstrate enhanced ability to generate horizontal forces during directional changes.^[12]^ This morphological advantage translates to reduced time requirements for center of gravity adjustments during rapid directional transitions, ultimately facilitating superior performance outcomes through improved biomechanical efficiency.^[12]^

Although evidence suggests that anthropometric characteristics affect change-of-direction speed performance, this relationship must be interpreted within the broader context of talent identification paradigms. Two contrasting perspectives dominate the discourse on talent selection: the innate talent hypothesis, which proposes that only individuals with genetic predispositions can achieve elite status, and the developmental hypothesis, suggesting that any individual can reach elite levels through dedicated practice and training.^[8]^ These perspectives converge on the principle that talent identification should occur early in development, with children being directed toward activities that match their inherent capabilities. During adolescence, young players experience significant variations in growth and biological maturity that substantially complicate future performance prediction.^[11]^ Contemporary talent identification frameworks acknowledge that player development trajectories are nonlinear and that current performance must be understood within the context of individual growth patterns, biological maturation status, and environmental influences.^[13]^ This developmental complexity necessitates continuous modeling of relationships between measurable characteristics and performance outcomes to establish accurate evaluation criteria for training program design.

Machine learning and artificial intelligence algorithms have emerged as powerful analytical tools in sports sciences, supporting diverse applications including performance analysis, injury prevention and rehabilitation, competition analysis, athlete tracking and data collection, and sports management.^[14]^ Recent implementations of machine learning methods in talent prediction demonstrate the potential for these technologies to optimize future predictions and decisions by identifying hidden patterns within complex datasets.^[15]^ The capacity of machine learning applications to determine dataset complexity through multilayer neural networks and generate predictions based on derived findings suggests that these methodologies should be extensively utilized in motor characteristic identification and talent screening processes.^[16]^ Contemporary applications emphasize the potential for algorithms to manage inherent complexity and nonlinear relationships characterizing anthropometric-performance associations in youth athletes.^[17]^

Comprehensive studies examining anthropometric characteristics that determine speed performance using machine learning algorithms remain limited in the current literature. Previous investigations utilizing machine learning approaches for predicting anthropometric-performance have produced inconsistent findings, highlighting the need for a systematic evaluation of different algorithms and their relative effectiveness in youth populations.^[15]^ Furthermore, the absence of explainable artificial intelligence approaches represents a significant limitation, as traditional machine learning models often operate without revealing their underlying decision-making processes.

This investigation aims to predict morphological features that determine change of direction speed performance in youth soccer players through machine learning applications, addressing critical gaps in the current understanding. Our research hypothesis states that specific morphometric characteristics significantly affect change of direction speed performance in youth soccer players, with biological maturation status and anthropometric parameters serving as primary determinants.

## 2. Methods

### 2.1. Study design

This cross-sectional study employed a machine learning approach to investigate the anthropometric predictors of change-of-direction speed performance in youth soccer players. The research protocol received approval from the Institutional Review Board of the University of Manouba (Ethics Committee Reference: LR23JS01-2023). All procedures adhered strictly to the principles outlined in the Declaration of Helsinki for human research. Written informed consent was obtained from parents or legal guardians, and participant assent was secured from all youth athletes before data collection. Participants and their guardians received comprehensive information regarding the experimental protocols, potential risks, and benefits before the study commenced.

### 2.2. Participants

Fifty-one elite male youth soccer players were recruited from a professional academy using purposive sampling. Inclusion criteria comprised: male soccer players aged 10 to 15 years, minimum 4 years of structured training experience, current participation in competitive leagues, absence of musculoskeletal injuries within 6 months preceding testing, and completion of all anthropometric and performance assessments. Exclusion criteria included: recent lower limb injuries, concurrent participation in other sports, incomplete data sets, and inability to perform maximal effort testing. Following quality assessment, 42 participants (age: 12.11 ± 1.23 years; body mass: 39.05 ± 7.22 kg; height: 1.51 ± 0.11 m) comprised the final analytical sample for machine learning modeling. Of the initial 51 athletes, 9 were excluded due to a data quality issue (missing data).

The initial cohort (n = 51; age: 11.88 ± 1.38 years; body mass: 38.25 ± 7.89 kg; height: 1.51 ± 0.11 m) participated in reliability testing. Following data quality assessment, 42 participants (age: 12.11 ± 1.23 years; body mass: 39.05 ± 7.22 kg; height: 1.51 ± 0.11 m) comprised the final analytical sample for machine learning modeling. Sample size determination was based on established guidelines for machine learning applications in sports science, requiring a minimum of 10 to 15 observations per predictor variable to ensure model stability and generalizability.^[18]^

All participants maintained consistent training schedules, comprising 4 to 5 sessions per week (7 ± 1 hours total) over the preceding 4 years. Training periodization was followed using structured progressive overload principles under the supervision of qualified coaches.

### 2.3. Anthropometric assessments

Anthropometric measurements were taken using standardized International Society for the Advancement of Ki anthropometry (ISAK) protocols.^[19]^ All assessments were conducted by a certified Level 2 ISAK anthropometrist, assisted by trained personnel, ensuring inter-observer reliability and measurement consistency. Instruments underwent calibration verification before each testing session.

Skinfold thicknesses were measured using precision Harpenden calipers (Harpenden Instruments, Cambridge, UK) at 4 anatomical sites on the body’s right side: biceps, triceps, subscapular, and supra-iliac. Three consecutive measurements were recorded at each site, with the median value retained for analysis.

Linear anthropometric dimensions were assessed using non-elastic anthropometric tape following landmark-based protocols. Standing height was measured from the floor to the vertex using a portable stadiometer. Sitting height represented the vertical distance from the seated surface to the vertex, reflecting trunk length. Leg length was calculated by subtracting sitting height from standing height, thereby providing an estimation of the subischial leg segment.

Circumferential measurements included calf girth at maximum lower leg circumference and thigh girth at the midpoint between the inguinal crease and the superior patellar border. Thigh length was measured from the inguinal ligament midpoint to the proximal patellar edge. Knee height represented the perpendicular distance from the anterior distal thigh surface to the floor.

Proportionality indices were computed, including: relative subischial leg length (RSLL = [(height − sitting height)/height] × 100), sitting height ratio (SHR = [sitting height/height] × 100), and knee height ratio (KHR = [knee height/height] × 100). These ratios provide indicators of segmental body proportions and biological maturation status.

### 2.4. Biological maturity assessment

Maturity offset was estimated using the validated predictive equation developed by Mirwald et al^[20]^: Maturity offset = −9.236 + 0.0002708 × (leg length × sitting height) − 0.001663 × (age × leg length) + 0.007216 × (age × sitting height) + 0.02292 × (weight/height) × 100. This noninvasive method provides reliable estimates of maturation in youth populations.

### 2.5. Change of direction speed testing

**Figure 1.**
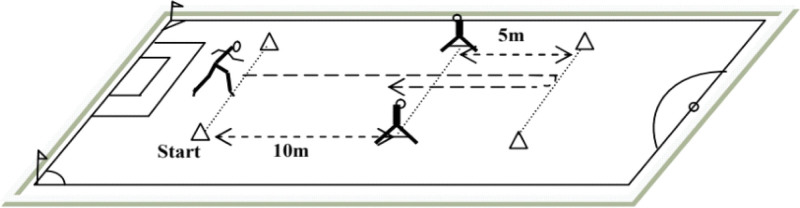
505 CoDS test protocol. Participants accelerated from a starting line and sprinted forward to a turning line located 15 m away, touching the line with 1 foot before rapidly changing direction and sprinting back 5 m through the timing gate. The test was repeated 3 times, with the fastest trial recorded for analysis. CoDS = change of direction speed.

The 505-test protocol followed standardized procedures established by Draper and Lancaster.^[21]^ Testing occurred on artificial turf during the early competitive season under consistent environmental conditions. Participants completed structured dynamic warm-up protocols before testing (Fig. 1).

Each participant performed 3 maximal-effort trials with 3-minute passive recovery intervals to minimize fatigue accumulation. Sprint times were recorded using high-precision electronic timing gates (Microgate, Bolzano, Italy) positioned at the point of turn. The fastest trial time was retained for subsequent analysis, consistent with established performance testing recommendations.^[22]^

Testing was conducted at least 48 hours posttraining or competition to control for residual neuromuscular fatigue effects, ensuring valid performance measurement.

### 2.6. Statistical analysis

Statistical analyses were performed using R version 4.4.3 (R Core Team, 2023) and SPSS Statistics version 27.0 (IBM Corp., Armonk). Data normality was assessed using Shapiro–Wilk tests. Test–retest reliability was evaluated using intraclass correlation coefficients (ICC: 3,*k*) and standard error of measurement (SEM), with statistical significance set at α = 0.05 (*P* < .05 considered significant).

Five tree-based regression algorithms were implemented: decision tree regression, random forest regression, extra trees regression, AdaBoost regression, and gradient boosting regression.^[5,18]^ For regression tree visualization, the dataset was randomly split into training (80%) and testing (20%) subsets; A decision tree was derived from the training set, while the reserved test set was used exclusively to evaluate predictive accuracy. Leave-one-out cross-validation was employed to maximize data utilization while minimizing overfitting bias.

Model performance was evaluated using the root mean square error (RMSE), coefficient of determination (*R*^2^), and Pearson correlation coefficient. Variable importance was assessed using Gini importance indices, with threshold values of 0.05 or below considered negligible. Missing data were handled through listwise deletion, given the availability of the complete dataset. Statistical significance was established at an α level of 0.05. In the predictive modeling framework, emphasis was placed on performance metrics and validation procedures rather than p-values.

## 3. Results

### 3.1. Performances on the 505-test test and retest

Residual data from the 505-test trials demonstrated normal distribution characteristics (Shapiro–Wilk test: *P* = .72 for the initial test, *P* = .56 for the retest), satisfying the parametric statistical assumptions. Dependent *t*-test analysis revealed no systematic bias between testing sessions (*P* = .60), with the estimated effect size indicating a trivial magnitude (Cohen *d* = 0.05) for all 505-test outcomes, confirming measurement stability across repeated assessments (Table 1).

**Table 1:** Performance characteristics of 505-test for young soccer players. Values are mean ± SD (N = 51).

	Test (Mean ± SD)	Retest (Mean ± SD)	*P*	*d*_z_
**505-test (s**)	2.64 ± 0.21 s	2.63 ± 0.17 s	.60	0.05

Data processing: Descriptive statistics and paired *t*-test for test–retest comparison.

505-test = 505 change of direction speed test, *d*_*z*_ = Cohen *d* effect size, SD = standard deviation.

### 3.2. Reproducibility of performance score on the 505-test

The 505-test demonstrated excellent relative reliability (ICC = 0.86) and superior absolute reliability characteristics. The standard error of measurement percentage was 3.54%, indicating precise measurement capability within acceptable thresholds for field-based performance testing. These reliability metrics established the 505-test’s sensitivity as appropriate for detecting meaningful performance variations in youth soccer populations (Table 2).

**Table 2:** Results of relative and absolute reliabilities of 505-test for young soccer players (N = 51).

	Relative reliability	Absolute reliability			
	ICC	SEM (s)	SEM% (%)	SWC_0.2_ (s)	SWC_0.6_ (s)
505-test	0.86	0.09	3.54	0.04	0.11

Data processing: Reliability analysis using ICC (3,*k*) and SEM calculation.

505-test = 505-test of speed of change of direction test, ICC = intraclass correlation coefficient, SEM = standard error of measurement, SWC = smallest worthwhile change.

### 3.3. Predicting change of direction speed performance

Comprehensive data collection encompassing physical performance, anthropometric measurements, skinfold assessments, and body composition variables provided the foundation for predictive modeling analyses. Algorithm comparison revealed substantial performance differences across regression methodologies, with decision tree regression demonstrating superior predictive accuracy and lower error rates for estimating change of direction speed in young athletes (Tables 3 and 4).

**Table 3:** Overall physical and anthropometric features (N = 42).

Variable	Q1	Mean	SD	Median	Q3
CoDS	2.48	2.61	0.19	2.59	2.76
Age	10.9	12.1	1.23	12.2	13.3
BM	32.8	39.1	7.22	38.2	45.2
BMI	15.4	16.5	1.60	1.64	17.6
BSK	4.00	5.00	1.72	4.75	5.95
CG	28.2	29.5	2.33	30	31
Ht	1.44	1.52	0.11	1.52	1.60
KHR (%)	24.00	25.2	1.70	25.30	26.10
KHt	36.00	38.40	3.68	38.00	39.80
LL	80.00	90.20	13.00	86.50	102.00
MO	−2.61	−1.93	0.97	−2.20	−1.17
PHV	13.60	14.00	0.54	14.00	14.50
RSLL (%)	51.80	52.90	1.99	53.00	53.80
SHR	46.20	47.10	1.99	47.00	48.20
SHeight	0.68	0.72	0.05	0.71	0.75
SISk	4.25	5.38	1.66	5.00	5.98
SSSk	4.82	5.67	1.26	5.30	6.07
STSk	11.00	13.10	3.22	12.20	14.20
TSk	5.08	7.16	2.29	7.10	8.78
ThG	39.00	41.40	3.00	41.00	43.00
ThL	37.00	40.80	4.98	39.00	43.80

Data processing: descriptive statistics (mean, median, quartiles, SD).

BM = body mass, BMI = body mass index, BSK = bicipital skinfold, CG = calf girth, CoDS = change of direction speed, Ht = height, KHR = knee height ratio, KHt = knee height, LL = leg length, MO = maturity offset, PHV = peak height velocity, Q1 = first quartile, Q3 = third quartile, RSLL = relative subischial leg length, SD = standard deviation, SHeight = sitting height, SHR = sitting height ratio, SISk = supra-iliac skinfold, SSSk = subscapular skinfold, STSk = sum of tricipital and subscapular skinfolds, ThG = thigh girth, ThL = thigh length, TSk = tricipital skinfold.

**Table 4:** Attained performance of the sprint prediction model using all input features.

	RMSE	*R* ^2^	MAE
Decision tree regression	0.072	0.815	0.065
Random forest regression	0.085	0.780	0.078
Extra tree regression	0.091	0.807	0.078
AdaBoost regression	0.095	0.743	0.075
Gradient boosting for regression	0.096	0.706	0.093

Data processing: model performance evaluation using cross-validation.

DTR = decision tree regression, MAE = mean absolute error, *R*^2^ = coefficient of determination, RMSE = root mean square error.

**Figure 2.**
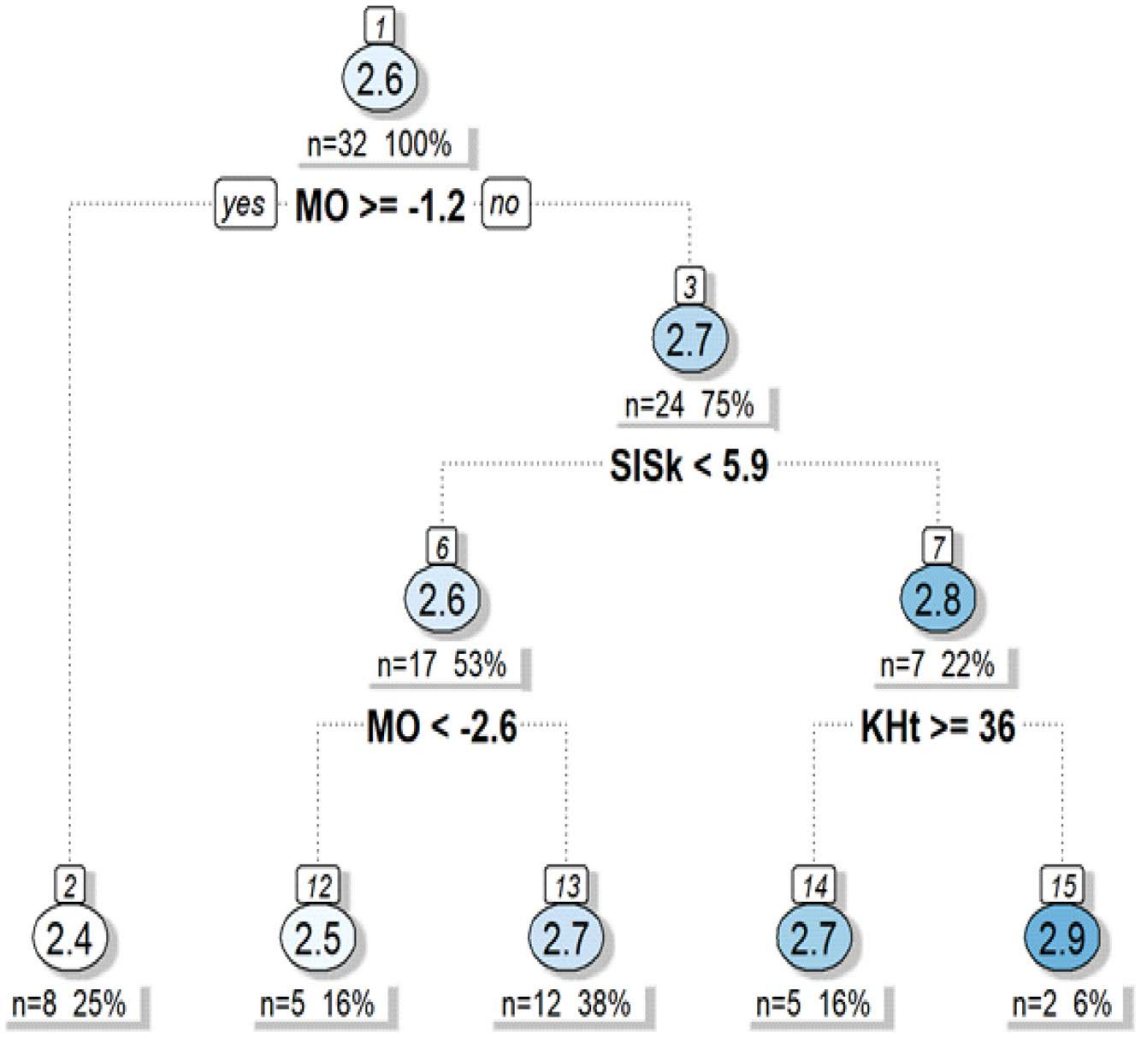
Decision tree model for predicting CoDS performance. Decision tree regression generated from the training dataset (80% of the total analytical sample, n = 32) using the classification and regression tree (CART) algorithm. The model stratifies CoDS performance outcomes based on maturity offset and key anthropometric predictors. Node values indicate mean predicted CoDS times, with sample sizes (n) and relative proportions (%) reported below each split. The hierarchical tree structure illustrates the sequential decision rules leading to performance predictions, with optimal CoDS performance (≤2.4 s) associated. CoDS = change of direction speed.

The optimized decision tree regression model, applied to the test dataset, yielded exceptional performance metrics: RMSE of 0.133, *R*^2^ of 0.843, and MAE of 0.111. Variable importance analysis identified 6 primary predictors contributing most substantially to model performance. Maturity offset emerged as the leading predictor (16.3%), followed closely by chronological age (16.3%), reflecting the fundamental influence of biological development on neuromuscular capabilities. Leg length contributed 15.8% to predictive accuracy, while sitting height accounted for 13.6% of model variance, emphasizing the biomechanical significance of lower limb proportions in directional change tasks. Body mass (10.1%) and knee height (10.5%) provided additional predictive value, completing the core anthropometric profile determining change of direction speed performance (Table 5, Fig. 2).

**Table 5:** The importance of each independent variable.

Dependent variable	Model	RMSE	*R* ^2^	Predictor	Importance			Linear regression	
					Inc node purity	GI%	Entropy	Coefficient	SSE
**CoDS**	DTR	0.07	81.5%	MO	0.79	16.3	2.79	−0.872	0.007
				Age	0.77	16.8	2.81	0.284	0.065
				LL	0.70	15.8	2.80	−0.007	0.047
				SHeight	0.65	13.6	2.94	10.997	0.069
				KHt	0.54	10.5	2.81	−0.009	0.020
				BM	0.51	10.1	2.75	−0.001	0.0002

Data processing: feature importance analysis using decision tree regression. With advanced biological maturity and favorable body segment proportions.

BM = body mass, CoDS = change of direction speed, GI% = Gini importance percentage, KHt = knee height, LL = leg length, MO = maturity offset, SHeight = sitting height, SSE = standard error of estimate.

The decision tree architecture revealed hierarchical performance stratification based on interactions between maturation and anthropometry. Participants with advanced biological maturity (maturity offset ≥ −1.2 years) consistently achieved superior performance times, approaching 2.4 seconds, while those exhibiting delayed maturation, combined with unfavorable anthropometric characteristics, demonstrated completion times exceeding 2.9 seconds. This stratification pattern underscores the complex interplay between developmental status and morphological traits in determining athletic performance capabilities during adolescence. The model’s capacity to explain 84.3% of performance variance through these 6 predictors demonstrates the substantial influence of biological and anthropometric factors on change of direction speed, providing valuable insights for talent identification and development practices in youth soccer programs.

## 4. Discussion

This investigation demonstrates that decision tree regression surpassed random forest regression, extra trees regression, AdaBoost regression, and gradient boosting regression in detecting the importance of selected anthropometric and demographic features for predicting change of direction speed performance in youth soccer players. The superior performance of our DTR model, explaining 81.5% of the variance in change of direction speed performance, establishes a new benchmark for morphological prediction accuracy in youth athletic populations.

Change of direction speed represents a fundamental physical attribute in field-based team sports, with soccer analyses demonstrating that rapid directional movements occur frequently (every 2 to 3 seconds) and repeatedly (>1000 per game) throughout competitive play.^[23,24]^ Contemporary research consistently identifies the change of direction speed as a distinguishing factor between elite and subelite youth soccer players, establishing its relevance for talent identification processes.^[2,3]^ Our DTR model achieved exceptional predictive accuracy (*R*^2^ = 0.843, RMSE = 0.133), substantially exceeding established thresholds in sports machine learning applications. This performance contrasts notably with the findings reported by Bongiovanni et al,^[25]^ where extra trees regression demonstrated superior predictive capability for assessing the change-of-direction deficit. However, their ETR model achieved only *R* = 0.33, failing to reach their predetermined significance threshold of *R* = 0.47. The discrepancies between our findings and those of Bongiovanni et al^[25]^ likely stem from fundamental differences in the variable selection strategies employed. Specifically, Bongiovanni et al^[25]^ calculated the change of direction ability using the “change of direction deficit,” defined as the time difference between a straight-line sprint and a change-of-direction task. This approach isolates deceleration and reacceleration components, but may overlook holistic movement execution relevant in sport-specific settings. In contrast, our study utilized the absolute time to complete the 505-test as the dependent variable, which reflects an integrated measure of neuromuscular control, force production, and biomechanical efficiency during a standardized directional change task. While our investigation focused primarily on lower limb anthropometric measurements and derived parameters alongside standing height, sitting height, and BMI, Bongiovanni et al^[25]^ incorporated both upper and lower limb anthropometric indices, including corrected arm muscle area, waist circumference, fat mass, arm muscle circumference, fat-free mass, and hip circumference.

The DTR model identified 6 primary predictors with substantial importance values: maturity offset (16.3%), chronological age (16.8%), leg length (15.8%), sitting height (13.6%), knee height (10.5%), and body mass (10.1%). These findings align with theoretical frameworks proposed by Sheppard and Young,^[26]^ which position anthropometric indices as critical subcomponents influencing change of direction speed in team sport athletes. Previous investigations by Negra et al^[1]^ established maturity offset as the most significant predictor of 505 change of direction performance in male youth soccer players, findings corroborated by our variable importance analysis. Chronological age emerged as one of the most influential variables, with participants under 13 years consistently demonstrating slower change of direction speed performance. This age-related improvement pattern reflects the nonlinear development trajectory documented throughout childhood and adolescence.^[7,27]^ Our findings contradict those of Bongiovanni et al,^[25]^ whose ETR model revealed no substantial age-related effects when the change of direction was expressed as deficit values, highlighting methodological differences in performance quantification approaches.

Body mass demonstrated significant negative associations with change-of-direction performance, confirming previous observations by Negra et al^[1]^ and Chaouachi et al.^[6]^ This relationship reflects fundamental biomechanical principles whereby optimal change of direction speed requires lower body mass and greater relative strength to generate the mechanical forces necessary for rapid directional alterations,^[28]^ consistent with Newton laws of motion.^[29]^ Sitting height exhibited negative correlations with performance outcomes, supporting center of mass optimization theories in directional change tasks. This finding suggests that lower center of mass positions provide biomechanical advantages during change-of-direction tasks, enabling more efficient horizontal force application compared to athletes with elevated center of mass positions.^[30]^ Athletes with shorter trunk segments require less time to lower their center of gravity during rapid directional transitions, which facilitates superior performance outcomes compared to their taller counterparts.^[31]^ Expanding on the roles of the 6 primary predictors identified by the DTR model further highlights the multifaceted physiological and biomechanical factors influencing change of direction speed. Maturity offset and chronological age capture biological and developmental progression, which underpin neuromuscular adaptations critical for rapid deceleration and acceleration during directional changes.^[32]^ Leg length and knee height influence leverage and stride mechanics, contributing to an athlete’s ability to generate force quickly and efficiently during cuts and turns.^[33]^ Sitting height is closely related to trunk length and center of mass positioning, factors that affect balance and stability, which are essential for maintaining control during rapid directional shifts.^[34]^ Lastly, body mass plays a dual role. In contrast, sufficient muscle mass can enhance force production; excess mass, however, may hinder agility due to increased inertia, highlighting the importance of maintaining an optimal body composition.^[35]^ Together, these predictors reflect a complex interaction of growth, morphology, and neuromuscular control that drives high-level change-of-direction performance in youth soccer.^[36]^

Our classification and regression tree (CART) model approach provided a granular understanding of performance stratification patterns. The decision tree revealed that optimal change of direction performance (2.4 seconds) was consistently associated with athletes exhibiting advanced maturity offset (≥−1.2 years). In comparison, the poorest performances (2.9 seconds) were observed among participants with delayed maturation, combined with elevated supra-iliac skinfold thickness and extended knee height. This 0.5-second performance differential represents practically meaningful differences that may influence competitive outcomes and talent selection decisions. These findings reinforce previous evidence demonstrating substantial improvements in change of direction speed during childhood and adolescence, particularly during peak height velocity periods, which mark critical windows of neuromuscular development.^[1,37]^ The observation that both chronological age and maturity offset emerged as robust independent predictors supports the notion that biological maturation drives neuro-motor control, postural adjustments, and movement efficiency during high-intensity deceleration and reacceleration tasks. The hierarchical performance stratification suggests that morpho-functional imbalances, such as disproportionate trunk dominance or elongated lower limb segments, may compound biological immaturity limitations by disrupting balance and joint mechanics during directional transitions. Previous research by Negra et al^[1]^ demonstrated that players with advanced maturity outperformed less mature peers, despite matching for anthropometric parameters and chronological age. These findings are confirmed and extended in our investigation through the incorporation of additional morphological factors.

Modern applications of machine learning in sports science underscore the ability of algorithms to identify hidden patterns in complex athletic performance data. Our use of decision tree regression with leave-one-out cross-validation is a carefully designed approach to modeling morphological prediction, tackling common overfitting issues in small-sample sports studies. Achieving 84.3% explained variance with 6 easy-to-measure predictors shows strong predictive power while remaining practical for field assessments.

Several limitations must be acknowledged when interpreting these findings. The 505 change-of-direction test, although validated and reliable, may not capture all change-of-direction movement patterns encountered in soccer competition. Additionally, noninvasive maturity assessment methods, despite their accessibility and practical benefits, have inherent limitations when applied to young athletes and should be interpreted cautiously.^[1,38]^ Future research should validate these decision tree profiles across diverse populations and sports disciplines and explore how individualized training interventions can help mitigate morpho-maturational disadvantages in late-developing athletes. Moreover, studies should examine the stability of these anthropometric predictors over time during adolescent development and how they interact with training-induced adaptations.

### 4.1. Practical implications

These findings underscore the importance of integrated profiling approaches that combine biological, anthropometric, and functional assessments to guide training design, performance monitoring, and talent identification. Coaches should adopt maturationally sensitive and morphology-aware frameworks when assessing adolescent athletes, moving beyond single-metric evaluations toward comprehensive developmental profiling. The decision tree model provides practical guidelines for identifying athletes with optimal morpho-functional profiles, while also recognizing those who require targeted interventions to enhance change-of-direction capabilities. Encouraging the use of machine learning applications can help determine the importance of anthropometric features in predicting athletic performance, enabling practitioners to strategically monitor specific variables while enhancing change-of-direction skills in youth soccer development programs.

## 5. Conclusion

This study shows that explainable artificial intelligence can effectively interpret how anthropometric factors influence change of direction speed in youth soccer players, achieving high predictive accuracy with easily measurable physical variables. The results support maturation-aware talent identification methods and offer biomechanical insights into the morphological and functional factors that determine directional change ability. Future research should confirm these decision tree profiles across different populations and investigate how personalized training programs can reduce morpho-maturational disadvantages in youth athletes.

## Acknowledgments

The authors are grateful to coaches and players for their enthusiasm and commitment to the completion of this study.

## Author contributions

**Conceptualization:** Akram Fray, Ahmed Attia.

**Data curation:** Yassine Negra, Younes Hachana, Mevlut Yildiz.

**Formal analysis:** Halil İbrahim Ceylan, Valentina Stefanica, Mevlut Yildiz.

**Investigation:** Yassine Negra, Younes Hachana, Valentina Stefanica, Mevlut Yildiz.

**Methodology:** Akram Fray, Ahmed Attia, Younes Hachana.

**Project administration:** Halil İbrahim Ceylan.

**Resources:** Yassine Negra.

**Software:** Younes Hachana.

**Supervision:** Halil İbrahim Ceylan.

**Validation:** Ahmed Attia, Yassine Negra.

**Visualization:** Valentina Stefanica.

**Writing – original draft:** Akram Fray, Ahmed Attia.

**Writing – review & editing:** Younes Hachana, Halil İbrahim Ceylan, Valentina Stefanica, Ahmed Attia, Yassine Negra.
